# Surfactant Monolayer Bending Elasticity in Lipase Containing Bicontinuous Microemulsions

**DOI:** 10.3389/fchem.2020.613388

**Published:** 2021-01-05

**Authors:** Sandra Engelskirchen, Stefan Wellert, Olaf Holderer, Henrich Frielinghaus, Michaela Laupheimer, Sven Richter, Bettina Nestl, Bernd Nebel, Bernhard Hauer

**Affiliations:** ^1^Department of Chemistry, Universität Stuttgart, Stuttgart, Germany; ^2^Department of Chemistry, Technische Universität Berlin, Berlin, Germany; ^3^Jülich Centre for Neutron Science (JCNS) at Heinz Maier-Leibnitz Zentrum (MLZ), Forschungszentrum Jülich GmbH, Garching, Germany

**Keywords:** microemulsion, lipase, bending elasticity, neutron scattering, neutron spin echo

## Abstract

Lipase-catalyzed reactions offer many advantages among which a high degree of selectivity combined with the possibility to convert even non-natural substrates are of particular interest. A major drawback in the applicability of lipases in the conversion of synthetically interesting, non-natural substrates is the substantial insolubility of such substrates in water. The conversion of substrates, natural or non-natural, by lipases generally involves the presence of a water–oil interface. In the present paper, we exploit the fact that the presence of lipases, in particular the lipase from *Candida antarctica* B (CalB), changes the bending elastic properties of a surfactant monolayer in a bicontinuous microemulsion consisting of D_2_O/NaCl -n-(d)-octane-pentaethylene glycol monodecyl ether (C_10_E_5_) in a similar manner as previously observed for amphiphilic block-copolymers. To determine the bending elastic constant, we have used two approaches, small angle neutron scattering (SANS) and neutron spin echo (NSE) spectroscopy. The time-averaged structure from SANS showed a slight decrease in bending elasticity, while on nanosecond time scales as probed with NSE, a stiffening has been observed, which was attributed to adsorption/desorption mechanisms of CalB at the surfactant monolayer. The results allow to derive further information on the influence of CalB on the composition and bending elasticity of the surfactant monolayer itself as well as the underlying adsorption/desorption mechanism.

## 1. Introduction

Lipases belong to the most well-investigated class of enzymes. Lipases show positional, substrate, and stereo-specificity toward their substrates. Whether or not lipases show as well promiscuous activity depends on reaction conditions and the nature of the substrate. Recent research revealed that lipases show great potential in catalyzing standard organic reactions such as: *C-C bond formation, Aldol-addition, Michael-addition, Mannich reaction, C-heteroatom and heteroatom-heteroatom bond formation, perhydrolysis and epoxidation, synthesis of heterocycles, and Hantzsch reactions* (Dwivedee et al., 2018). The non-natural substrates used in these organic reactions are, however, usually poorly water soluble such that a vehicle needs to be found to bring the lipases into contact with these kind of substrates.

**Figure 1:**
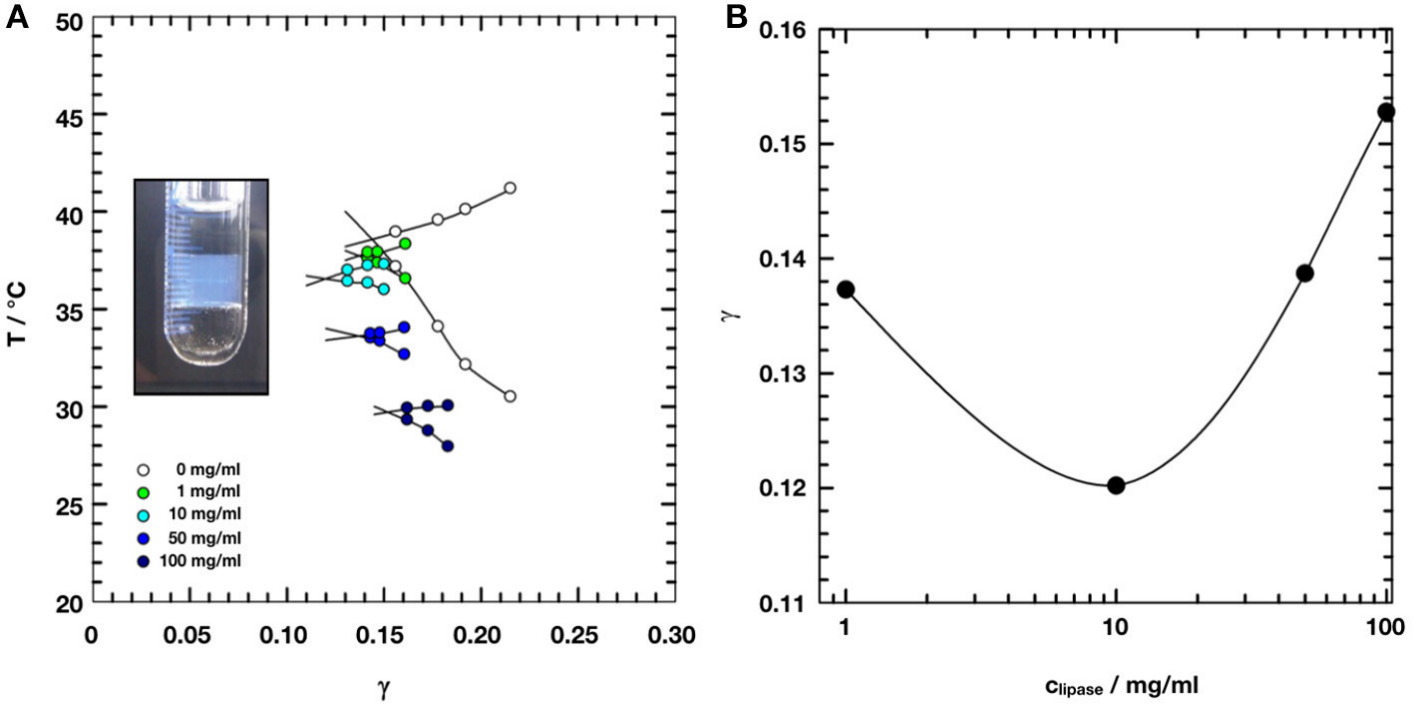
**(A)** Phase diagrams of microemulsions of type H_2_O/NaCl (0.4wt%)– *n*-octane–C_10_E_5_–lipase *Candida antarctica B* at equal volume fractions of water and oil ϕ = 0.50 with increasing concentration of lipase from 0 to 100 mg/ml referred to the water phase. Adapted with permission from Subinya et al. (2014), Copyright 2014 American Chemical Society. **(B)** X-points of the respective microemulsions as a function of lipase concentration. At 10 mg/ml of lipase, the efficiency of the microemulsion runs through a maximum.

Colloidal dispersions, especially microemulsions, have been identified to offer great potential acting as a reaction vehicle for lipase and substrate in providing large interfacial area between water and oil domains. In the present study, we exploit the fact that the influence of the lipase from *Candida antarctica B* (CalB) has already been investigated (Subinya et al., 2014). Figure 1A shows the phase diagrams obtained at equal volume fractions of sodium chloride solution (0.4 wt%) and *n*-octane (ϕ = 0.50) as a function of temperature and C_10_E_5_ mass fraction γ for increasing CalB concentrations added to the water phase: 1, 10, 50, and 100 mg/ml. Together with the minimum surfactant concentration needed for complete solubilization of water and oil in the onset of the single-phase region, the phase inversion temperature defines the point X (*T*_m_, γ). In the blank H_2_O/NaCl (0.4 wt.%)–*n*-octane–C_10_E_5_ system, the location of the X-point had already been well-known (*T*_m_ = 38.75°C, γ=0.15). Introducing increasing CalB concentrations to the water phase of the microemulsion revealed that for CalB concentrations of up to 10 mg/ml, the phase boundaries shifted to lower temperatures and the efficiency of the microemulsion increased, hence, less C_10_E_5_ was needed to totally solubilize the water and the octane phase. Above this concentration, the temperature trend continued, but the efficiency trend was found to be reversed. The efficiency even decreased below the blank microemulsion. In partitioning studies in Subinya et al. (2014), it has also been shown that the largest part, 80–90%, of CalB is related to the interface. Figure 1B shows the development of $\tilde{\gamma }$ as a function of CalB concentration to visualize the trends observed. Furthermore, it was found that the temperature *T*_*m*_ drops continuously with increasing CalB amount. CalB thus obviously induces the surfactant monolayer, which forms the microemulsion membrane, to bend toward the water domain.

To characterize the structural parameters and to access the bending elastic constants of an amphiphilic interface, scattering methods still remain the first choice. In recent years, many studies used light, X-ray, and neutron scattering techniques to explore the manifold of structures that are present in the different phase regions of the large variety of the studied phase systems (Komura, 2007; Gradzielski, 2008; Hoffmann, 2014; Oberdisse and Hellweg, 2017). Beside the influence of composition and thermodynamic variables, the changes in the phase structures due to the presence of additives, for example, enzymes, are accessible by applying these techniques, which improves the understanding of the bio-organic reaction mechanisms inside microemulsions (Hayes et al., 2015). For example, a combination of scattering techniques was used to investigate the effect of the enzyme DFPase, which efficiently hydrolyzes organophosphates, on the structure and dynamics of the host microemulsion with a bicontinuous structure (Wellert et al., 2011). Moreover, enzymes, such as the green fluorescent protein can be used as probes inside the aqueous phase of bicontinuous microemulsions to study fundamentals of diffusion in confinement (Wrede et al., 2019).

In this paper, we combine neutron small angle scattering (SANS) and neutron spin echo spectroscopy (NSE) to measure the effect of the addition of the lipase CalB on the amphiphilic interface inside the investigated bicontinuous microemulsions of the ternary system D_2_O/NaCl–n-(d)-octane–C_10_E_5_. Changes in the bending elastic constants observed upon the introduction of CalB at different concentrations up to 100 mg/ml provide deep insights into the mechanisms underlying the adsorption/desorption of the lipase to the interfacial layer.

## 2. Theory

Unlike emulsions and nanoemulsions, microemulsions are thermodynamically stable, fluid mixtures of at least water, oil, and surfactant (Strey, 1994; Schwarz and Gompper, 2002). Such mixtures may adopt a variety of complex structures depending on the nature of the components, their concentration in the mixture, and on temperature and pressure. The structural properties of the interface depend strongly on the surfactant packing parameter, which measures the ratio of the size of the hydrophilic headgroup and the length of the lipophilic alkyl chain. The topology of the amphiphilic interface is characterized by its curvature and mechanical properties.

The structure of bicontinuous microemulsions can be analyzed either by high-resolution electron microscopy on a freeze fractured microemulsion sample (Davis et al., 1989; Belkoura et al., 2004) or small angle neutron or X-ray scattering (Sottmann et al., 1997; Byelov et al., 2004; Oberdisse and Hellweg, 2017). Characteristic features of the bicontinuous structure are a low degree of ordering and an interconnected, random distribution of oil and water domains of varying size. In a bicontinuous microemulsion, the spontaneous curvature is zero, i.e., the bending of the surfactant film to either side requires the same amount of energy (Schwarz and Gompper, 2002). Cryo TEM images of a very similar bicontinuous microemulsion can be found (e.g., in Belkoura et al., 2004). Typically, such samples show scattering patterns with a broad peak and a steep decay of the scattered intensity at higher scattering angles, respective higher values of the scattering vector Q.

If the sample is prepared in bulk contrast, which provides high scattering contrast between the protonated oil and the deuterated water phase, the scattered intensity can be described by the *Teubner–Strey* (Teubner and Strey, 1987) equation:

(1) $$\begin{array}{l} I_{\text{TS}}\left(Q\right)=\frac{8\pi \langle \nu^{2}\rangle /\xi_{\text{TS}}}{p^{2}-2Q_{\text{max}}^{2}Q^{2}+Q^{4}} \end{array}$$

In this model, Q is the magnitude of the scattering vector defined as *Q* = 4π/λ sin(θ/2) with the scattering angle θ and 〈ν^2^〉 being the mean square scattering length density fluctuation, which is a measure of the scattering contrast. The quantities $p^{2}={\left(2\pi /D_{\text{TS}}\right)}^{2}+1/\xi_{\text{TS}}^{2}$ and $Q_{\text{max}}^{2}={\left(2\pi /D_{\text{TS}}\right)}^{2}-1/\xi_{\text{TS}}^{2}$ can be used to calculate the mean size of the oil and water domains D_TS_ and the mean correlation length ξ_TS_ of this structure.

The contribution of the elastic properties of the amphiphilic interface of a microemulsion to the free energy can be described by the *Helfrich* Hamiltonian:

(2) $$\begin{array}{l} f_{\text{el}}=\frac{1}{2}\kappa {\left(H-c_{0}\right)}^{2}+\bar{\kappa }K \end{array}$$

with the total curvature *H* = *c*_1_ + *c*_2_ of the film and *K* = *c*_1_*c*_2_ being the Gaussian curvature. This equation connects the bending elasticity constant κ and the saddle-splay modulus $\bar{\kappa }$ with the principal curvatures c_1_ and c_2_ and the mean curvature c_0_ of the interfacial surfactant layer in its lowest energetic state (Safran, 1999) taking into account not only elastic but also topological features of the respective structure formed by the amphiphilic interface. Here, the bending elasticity constant κ measures the energy required to deviate the interfacial film from the spontaneous curvature c_0_ at the free energy minimum. Similarly, the saddle-splay modulus $\bar{\kappa }$ is a measure of the energy cost for saddle-splay deformations (Safran, 1999). The bending elasticity constant is sensitive to changes in topology and interfacial composition and directly accessible by scattering. Thermally excited undulations lead to a softening of the interfacial film and to a length scale-dependent modification of the bare bending rigidity κ_0_, which is called the renormalized bending elasticity constant:

(3) $$\begin{array}{l} \frac{\kappa_{\text{SANS}}}{k_{\text{B}}T}=\frac{\kappa_{0,\text{SANS}}}{k_{\text{B}}T}-\frac{3}{4\pi }\ln\left(\frac{D_{\text{TS}}}{2l_{\text{s}}}\right) \end{array}$$

It is related to the structural length scales inside the bicontinuous structure by Pieruschka et al. (1995):

(4) $$\begin{array}{l} \frac{\kappa_{\text{SANS}}}{k_{\text{B}}T}=\frac{10\pi \sqrt{3}}{64}\frac{\xi_{\text{TS}}}{D_{\text{TS}}} \end{array}$$

The saddle-splay modulus can be obtained from phase diagram measurements (Holderer et al., 2013). We denote the bare moduli, which are not affected by renormalization effects, with the subscript 0, and denote the technique also in the subscript (which mainly is relevant for the renormalized moduli and only in the special case of influences of the observation time is also relevant for the bare moduli, as explained later). Monte-Carlo simulations of triangulated surfaces (Peltomäki et al., 2012) and combined phase diagram, SANS, and NSE experiments (Holderer et al., 2013) showed that the bending rigidity measured by SANS is a combination of κ and $\bar{\kappa }$, $\kappa_{0,SANS}=\left(a_{1}\kappa_{0}+a_{2}|{\bar{\kappa }}_{0}|\right)$. Also the renormalization term in Equation (3) is slightly modified by a combination of the renormalization terms of κ and $\bar{\kappa }$ and reads (3*a*_1_ + 10/3|*a*_2_|)/(4π)ln(*D*_*TS*_/2*l*_*s*_) with *a*_1_ = 0.19 and *a*_2_ = −0.84 (Holderer et al., 2013). These corrections have been applied for the values of the bending rigidity from SANS measurements presented in this paper.

Contributions to dynamic properties in bicontinuous microemulsions result from collective hydrodynamic motions of the sponge-like structure (Holderer et al., 2007) and local thermally excited undulations of the interfacial film (Safran, 1999). The bending elasticity constant κ of the interfacial film and the viscosity η of the surrounding solvent dissipate the undulation energy.

Neutron spin-echo spectroscopy measures the intermediate scattering function S(Q,τ_NSE_) normalized by the static structure factor S(Q,0) obtained from an appropriate reference sample. In the low Q-range, at Q < Q_max_, collective motions are dominant while with increasing Q the amplitude of these motions decreases until at Q>>Q_max_ the thermal undulations of the interface dominate. Zilman and Granek (1996) derived a stretched exponential form of the normalized intermediate scattering function *S*(*Q*, τ_NSE_)/*S*(*Q*, 0) as according to the following equation:

(5) $$\begin{array}{l} \frac{S\left(Q,\tau_{\text{NSE}}\right)}{S\left(Q,0\right)}=\exp\left(-{\left(\Gamma \tau_{\text{NSE}}\right)}^{\beta }\right) \end{array}$$

with a stretching exponent β = 2/3. The underlying model assumes an ensemble of randomly oriented and independently fluctuating membrane patches dispersed in the viscous solvent. Within this model, the relaxation rate of the membrane undulations is given by the following equation:

(6) $$\begin{array}{l} \Gamma_{u}=0.025\gamma_{\kappa }{\left(\frac{k_{B}T}{\kappa_{0,\text{NSE}}}\right)}^{1/2}\frac{k_{B}T}{\eta }Q^{3} \end{array}$$

which contains the bare bending elasticity constant κ_0, NSE_ of the interface and the parameter γ_κ_ given by γ_κ_ ≃ 1 − 3(*k*_B_*T*/(4πκ_0,NSE_)ln(*Qξ*) ≃ 1 for large enough κ. In a more recent paper, Zilman and Granek refined their analyses of the intermediate scattering function and deduced a more complex formulation including deviations of β from 2/3 depending on the bending elasticity constant (Zilman and Granek, 2002). However, since these effects are rather small, due to limited statistics and measurement time in a typical neutron scattering experiment, we use the previously derived exponent.

## 3. Materials

Pentaethylene glycol monodecyl ether (C_10_E_5_) was obtained in a purity of >97% from Sigma Aldrich, Germany. The lipase *Candida antarctica B* (CalB) was ordered in lyophilized form from Biocatalytics, Pasadena, USA. Purification was performed using ion-exchange chromatography and carried out in the Institute of Technical Biochemistry, Stuttgart, Germany, following the protocol given below. Sodium chloride p.a. was purchased from Merck, Germany, and n-octane in analytical grade was purchased from Fluka, Germany. D_2_O and d(18)-octane were obtained from Sigma Aldrich, Germany.

### 3.1. Purification Protocol

CalB was purified using a cation-exchange chromatography system. A XK 16/20 column (Amersham Biosciences) filled with 7 mL Source 15 S (Amersham Biosciences) was equilibrated with 10 mM sodium formate, 10 mM sodium citrate, and 10 mM sodium acetate, pH 3 (buffer A). A sample volume of 2 mL of the enzyme was loaded onto the column at a volumetric flow rate of 5 mL/min. Afterward, the column was washed with a threefold column volume of buffer A. CalB was eluted with five column volumes of 10 mM sodium formate, 10 mM sodium citrate, and 10 mM sodium acetate, pH 5.5. Chromatography columns and resins were purchased from Amersham Biosciences. All chromatography experiments were carried out at room temperature (20°C) using an ÅKTA explorer chromatography system (Amersham Biosciences) controlled by Unicorn software 3.21.

## 4. Experimental Section

### 4.1. Microemulsion Sample Preparation

All samples were prepared at a constant oil to water plus oil volume fraction of (ϕ) = 0.50:

(7) $$\begin{array}{l} \phi =\frac{V_{\text{oil}}}{V_{\text{oil}}+V_{\text{buffer}}} \end{array}$$

A mass fraction ϵ of 0.004 (0.4wt.%) of sodium chloride in D_2_O was kept constant for all samples:

(8) $$\begin{array}{l} \epsilon =\frac{m_{\text{NaCl}}}{m_{\text{NaCl}}+m_{\text{buffer}}} \end{array}$$

Each sample was prepared at a surfactant mass fraction of γ = 0.15 (15 wt.%) irrespective of the CalB concentration.

(9) $$\begin{array}{l} \gamma =\frac{m_{\text{surfactant}}}{m_{\text{surfactant}}+m_{\text{oil}}+m_{\text{buffer}}} \end{array}$$

This value was chosen from the previously obtained phase diagrams for microemulsions consisting of H_2_O/NaCl–n-octane–C_10_E_5_–CalB shown in Figure 1 (Subinya et al., 2014). This value was chosen to ensure that a single phase bicontinuous microemulsion would exist for every CalB concentration, thus taking into account the development of the efficiency of the microemulsion as a function of CalB concentration. In calculating sample compositions, the density differences of H_2_O and D_2_O as well as n-octane and d-octane were also taken into account.

All samples were prepared on site and phase boundaries were detected via visual inspection as a function of temperature. The respective components were weighed into glass vessels within an accuracy of ±0.0005 g, closed with a polyethylene stopper and sealed with Parafilm. A thermostated water bath setup consisting of thermostat (DC30, Haake, Germany), a precision thermometer (GMH 3750, Greisinger, Germany), a magnetic stirrer (HeiMix L, Heidolph, Germany), and a microscopy lamp (Gerhardt Optik and Feinmechanik, Germany) was used to obtain the phase boundaries. Phase transitions were determined to an accuracy of ±0.05°C.

The lipase CalB was introduced into the microemulsion from a concentrated stock solution of 100 mg/ml enzyme in D_2_O/NaCl solution and respective dilutions.

Different contrasts can be applied in neutron scattering by selectively deuterating parts of the sample. In this study, we measured the structure of the microemulsion in so-called “bulk contrast,” where H_2_O has been replaced by D_2_O, the rest being protonated materials. It provides contrast between the major domains water and (surfactant+oil). The SANS experiments have been performed in “bulk contrast.” For studying the membrane dynamics with NSE, “film contrast” has been chosen, with deuterated water, deuterated oil, and protonated the surfactant, providing contrast between the surfactant membrane and its environment. The enzyme was protonated in all cases. All experiments have been done close to the X-point of the phase diagram to ensure that temperature shifts due to deuteration are properly taken into account; the phase boundaries for each sample have been determined on site with the actual sample. After the determination of the phase boundaries, the samples were transferred into 2 mm Quartz cuvettes for NSE or 1 mm Quartz cuvettes for SANS measurements. Table 1 shows the respective sample measurement temperatures that were interpolated from the location of the phase boundaries as determined on site.

**Table 1:** Temperature conditions of the experiments.

**Lipase CalB (mg/ml)**	***T*_SANS_ (°C)**	***T*_NSE_ (°C)**
0	35.2	35.5
10	33.3	33.6
50	28.3	30.0
100	24.0	24.5

### 4.2. Small Angle Neutron Scattering

SANS measurements were performed on the small angle scattering instruments KWS-1 (Feoktystov et al., 2015; Frielinghaus et al., 2015) and KWS-2 (Radulescu et al., 2015) at the high flux neutron source FRM II (Garching, Germany). Scattering curves were determined at three different sample/detector (^6^Li-Scintillator 1 mm thickness + photomultiplier) distances of 2 m, 8 m, and 20 m accessing the q-range covering the structure peak followed by the *Q*^−4^ decay. Scattering measurements were performed at a neutron wavelength of λ = 4.7 Å (Dornier velocity selector, FWHM 10%). Quartz cuvettes of 1 mm in diameter (Hellma, Mülheim/Baden, Germany) were mounted on a thermostated sample holder. This sample thickness ensures that at the used wavelength multiple scattering effects play only a minor role in determining the structural parameters of the microemulsion (Frielinghaus, 2018). The temperature was operated by remote control to allow temperature-dependent measurements. Calibration measurements (empty cell, water, and plexi-glass) were carried out to adjust the spectrometer parameters. The raw data for the two detector distances were joined and the scattering signals were converted to absolute intensity using the supplied on-site software qtiKWS and qtiSAS. The resulting scattering curves were fitted to the *Teubner–Strey* model using Sigma Plot.

### 4.3. Neutron Spin-Echo Spectroscopy

In neutron spin-echo spectroscopy, the polarization state of a polarized neutron beam after quasi-elastic scattering at a sample is analyzed. The magnetic moment of the neutrons associated with the neutron spin is utilized to control and manipulate their Lamor precession in magnetic guiding fields prior and after the sample. The obtained polarization signal can be related to the Fourier transform of the dynamic structure factor S(Q,ω), which is the intermediate scattering function *S*(*Q*, τ_NSE_). The Fourier time τ_NSE_ is a parameter depending on the neutron wavelength and the integral of the magnetic field along the neutron path. It has the dimension of time. During a measurement, the Fourier time is varied by changing the magnetic field. Experimental details are well-described in the literature and we refer to a series of papers describing method and instrumentation (Hoffmann, 2014; Pasini et al., 2019; Zolnierczuk et al., 2019).

Neutron spin-echo measurements were carried out on the small angle spin-echo spectrometer J-NSE located at the NL2a neutron guide of the high flux neutron source FRMII (Garching, Germany)(Holderer et al., 2008). The normalized intermediate scattering functions *S*(*Q*, τ_NSE_)/*S*(*Q*, 0) were measured in a momentum transfer range of *Q* = 0.05–0.2 Å^-1^ using neutron wavelengths of 8, 10, and 12 Å exploring a Fourier time window of τ_NSE_ = 0.08–120 ns. However, here we focus on an analysis of the short time dynamics. Therefore, we restrict the measured data to Fourier times of up to 40 ns. The sample temperature was adjusted in a thermostated bath according to the previously determined phase behavior. The samples were prepared in film contrast and measured in Hellma Quartz cells of 2 mm thickness. The film contrast allows for thicker samples, and multiple scattering plays only a minor effect due to the weaker contrast and due to the circumstance that multiple incoherent scattering quickly depolarizes the beam.

## 5. Results

The aim of the presented study is to investigate the influence of the lipase CalB on the bending elasticity of a bicontinuous microemulsion with neutron scattering techniques. The symmetric microemulsion (with the same amounts of water and oil) was always held in the right temperature where it forms a sponge phase. The surfactant concentration was kept constant. Only the concentration of the lipase was varied as the main question, which shall be addressed here.

### 5.1. Determination of the Bending Elasticity Constant With SANS

**Figure 2:**
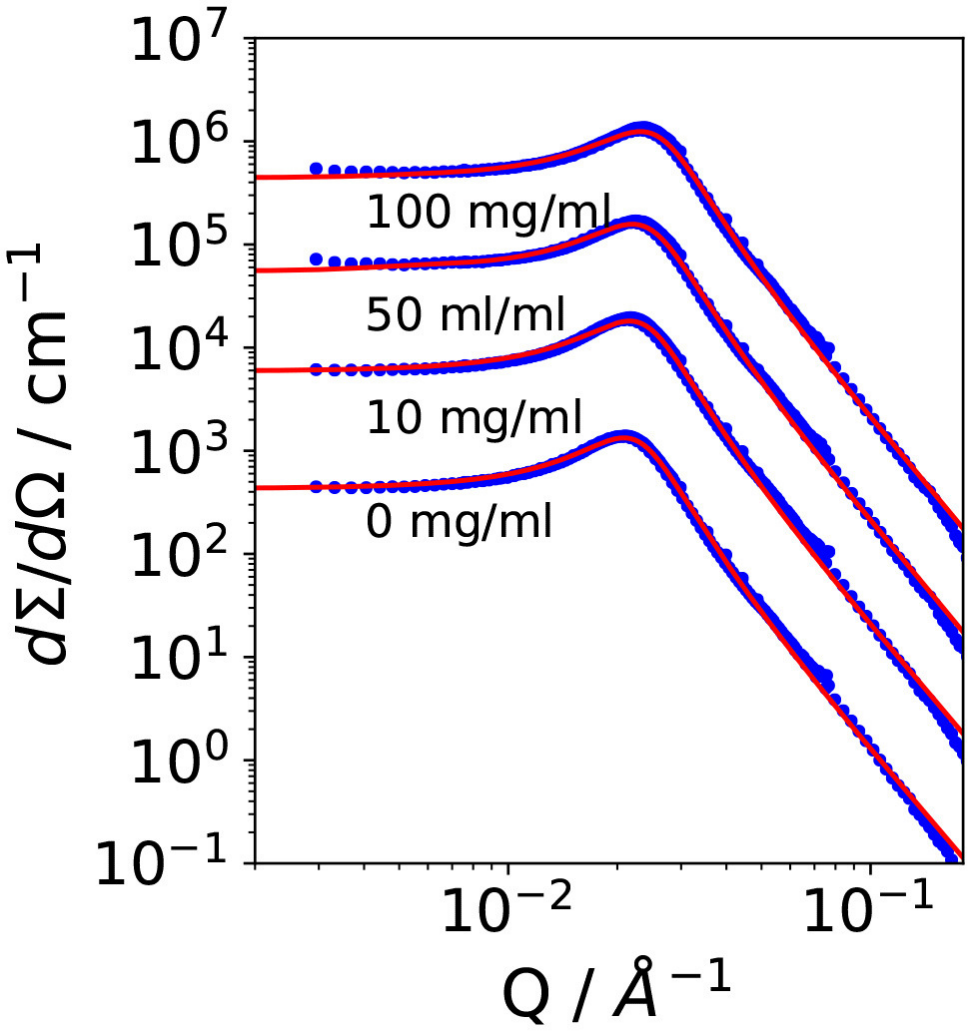
Small angle scattering data of the bicontinuous microemulsions from the ternary system H_2_O/NaCl–*n*-octane–pentaethylene glycol monodecylether (C_10_E_5_) with different amounts of lipase *Candida antarctica* B (CalB). The data were shifted by a factor of 10 for better visibility.

Figure 2 shows small angle scattering curves obtained for bicontinuous microemulsion samples containing no enzyme (reference, 0 mg/ml) and 10, 50, or 100 mg/ml of the lipase CalB.

All scattering curves show the characteristic plateau region in the low Q-range followed by the broad structure peak and the steep decay with a *Q*^−4^ dependence of the scattered intensity in the high *Q*-range. The solid lines are fits to the *Teubner-Strey* model, which describes the scattering around the structure peak. The Porod contribution at high-Q is included into the model (Frank et al., 2007):

(10) $$\begin{array}{l} I\left(Q\right)=\left(I_{\text{TS}}\left(Q\right)+\frac{G\;{\text{erf}}^{12}\left(1.06QR_{g}/\sqrt{6}\right)}{1.5Q^{4}R_{g}^{4}}\right)\exp\left(-\sigma^{2}Q^{2}\right)+I_{\text{bgr}} \end{array}$$

It is *R*_*g*_ the radius of gyration, σ a surface roughness parameter, and G the amplitude of the Porod scattering contribution. The calculated values of ξ_TS_ and *D*_TS_ are summarized in Table 2. Within the accuracy of the fit routine, no significant dependence of the values was observed when the concentration of CalB was increased up to 100 mg/ml.

**Table 2:** Parameters obtained from small angle neutron scattering (SANS) and phase diagram measurements: Correlation length and domain size determined from SANS experiments and deduced bending rigidity κ_SANS_, saddle-splay modulus ${\bar{\kappa }}_{0,Phase}$ from the phase diagram, and ${\bar{\kappa }}_{0,SANS}$ from SANS as well as κ_0,SANS|NSE_ from SANS and neutron spin echo (NSE) measurements as lined out in Holderer et al. (2013).

**Lipase CalB**	**ξ_TS_**	**D_TS_**	**κ_SANS_**	**${\bar{\kappa }}_{0,Phase}$**	**${\overset{¯}{\kappa }}_{0,SANS}$**	**κ_0,*SANS*_**	**κ_0,*NSE*_**
**(mg/ml)**	**(Å)**	**(Å)**	**(*k*_B_*T*)**	**(*k*_B_*T*)**	**(*k*_B_*T*)**	**(*k*_B_*T*)**	**(*k*_B_*T*)**
0	146	286	0.43	−0.69	−0.82	0.85	0.87 ± 0.03
10	140	276	0.43	−0.73	−0.79	0.84	0.92 ± 0.03
50	131	270	0.41	−0.69	−0.75	0.82	0.96 ± 0.02
100	124	256	0.41	−0.66	−0.72	0.80	1.03 ± 0.02

*The statistical errors from the fits were ≈1 Å for d and ξ, and 0.01 kT for the bending rigidities determined from SANS measurements*.

Following Safran and Pieruschka, the renormalized bending elastic constant κ_SANS_ can be calculated from *D*_TS_ and ξ_TS_ by applying Equation (4). Since the addition of CalB did not affect the structural parameters, the calculated values for κ_SANS_ remain constant as a function of CalB concentration.

### 5.2. Determination of the Bending Elasticity Constant From NSE

**Figure 3:**
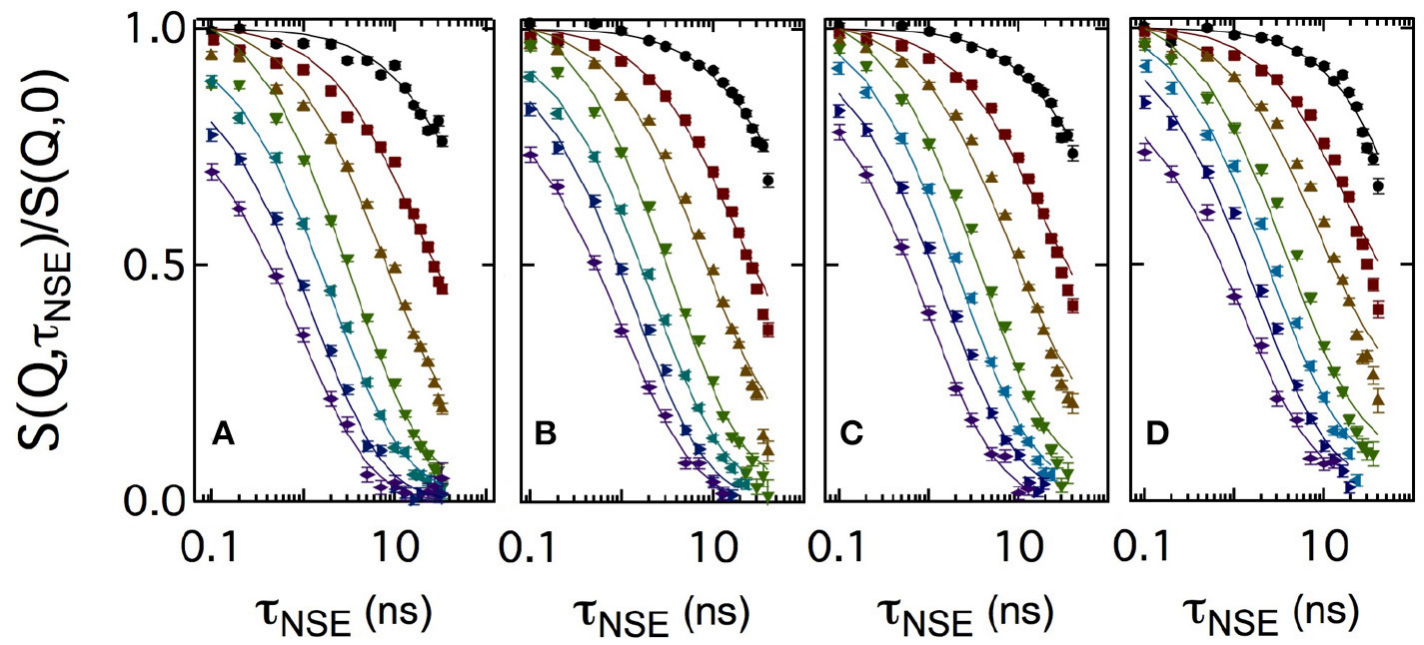
Semi-logarithmic representation of the normalized intermediate scattering functions *S*(*Q*, τ_NSE_)/*S*(*Q*, 0) obtained at momentum transfer values *Q* = 0.05, 0.08, 0.12, 0.15, 0.18, 0.21, and 0.24Å^-1^ (from top to bottom). A bicontinuous microemulsion in film contrast with four enzyme concentrations of 0 mg/ml **(A)**, 10 mg/ml **(B)**, 50 mg/ml **(C)**, and 100 mg/ml **(D)** was measured. The solid lines are fits to the data according to Equation (5).

Figure 3 shows a set of normalized intermediate scattering functions *S*(*Q*, τ_NSE_)/*S*(*Q*, 0) in semi-logarithmic representation. The data were measured with the bicontinuous microemulsions prepared in film contrast where in addition to the deuterated water phase also the oil phase was deuterated. As for the SANS measurements, CalB concentrations of 0 mg/ml (a), 10 mg/ml (b), 50 mg/ml (c), or 100 mg/ml (d) in deuterated sodium chloride solution were used. At low *Q* values, the curves show a partial decay. At larger *Q*, the curves fully decay to zero level within the observed Fourier time window. This is the general behavior observed for all measured lipase concentrations.

Usually, intermediate scattering functions measured with bicontinuous microemulsions are fitted with a combination of the stretched exponential accounting for the membrane undulations and a single exponential term including the collective hydrodynamic motions into the analysis. This collective dynamics is measured by dynamic light scattering in an additional independent experiment (Hellweg et al., 2001; Wellert et al., 2010; Klostermann et al., 2012). Here, we choose a different approach. At large Q, the diffusive component from large-scale density fluctuations may be neglected, because the Q^3^ dependence of the membrane fluctuations dominates at large Q over the Q^2^ dependence of the density fluctuations. A numerical computation routine of S(Q,τ_NSE_) was previously developed to avoid further approximations and complementary measurements:

(11) $$\begin{array}{l} S\left(Q,\tau_{\text{NSE}}\right)\propto \int_{0}^{1}d\mu \int_{0}^{R}dr\;rJ_{0}\left(Qr\sqrt{1-\mu^{2}}\right)\\ \exp\left(-\frac{k_{\text{B}}T}{2\pi \kappa }Q^{2}\mu^{2}\int_{k_{\text{min}}}^{k_{\text{max}}}\frac{dk}{k^{3}}\left(1-J_{0}\left(kr\right)e^{-\omega \left(k\right)t}\right)\right) \end{array}$$

which includes three nested integrals and the Bessel function of order 0 *J*_0_(*kr*). The integration limits are set by the maximum and minimum values of the system length scales. Here, the lowest wavevector k_min_ is related to the maximum real space value *r*_max_ = 2π/*k*_min_, with *k*_min_ = 2π/ξ and the upper limit *k*_max_ is given by *k*_max_ ≃ 2π/*a*. Bending rigidity was used as free parameter and at *Q* > 0.18Å^-1^ the amplitude becomes less than one due to background effects. Therefore, the amplitude normally fixed to 1 due to the normalization of *S*(*Q*, τ_NSE_)/*S*(*Q*, 0) was also used as free parameter. For a more detailed description of this numerical fitting approach, we refer to the literature (Mihailescu et al., 2001).

**Figure 4:**
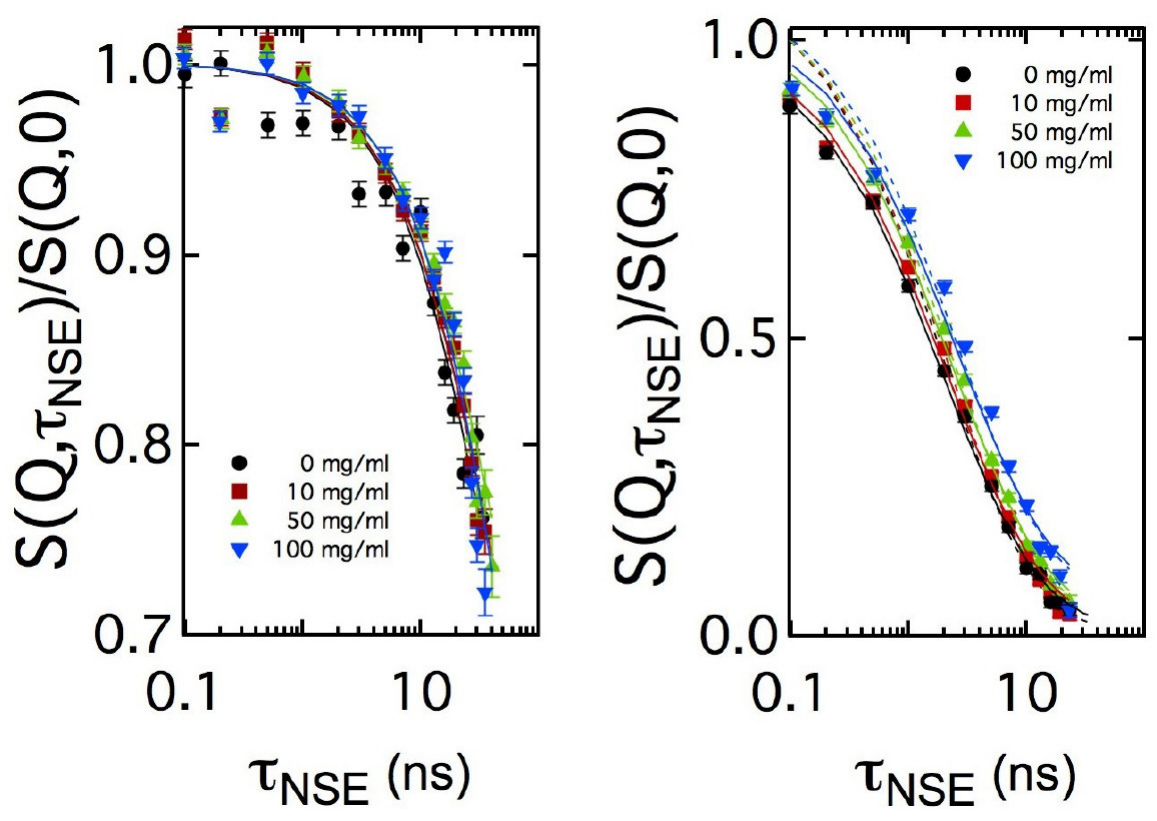
Comparison of the normalized intermediate scattering functions *S*(*Q*, τ_NSE_)/*S*(*Q*, 0) measured at *Q* = 0.05Å^-1^ and *Q* = 0.18Å^-1^ for all enzyme concentrations in semi-logarithmic representation. In the low Q-range, corresponding to length scales close to 2π/*Q*_max_, no significant difference in the decay rates is visible while at length scales much smaller than 2π/*Q*_max_ the decay of the intermediate scattering function slows down with increasing enzyme concentration. Solid lines are fits according to Equation (5). Additional dashed lines in the right image show fitting results with a fixed amplitude of *S*(*Q*, τ_NSE_)/*S*(*Q*, 0) = 1, while the solid line uses this amplitude as additional free fitting parameter.

For sake of comparison, in Figure 4 the curves measured at two *Q* values are plotted for the four CalB concentrations. At *Q* = 0.05Å^-1^, no significant difference in the decay of *S*(*Q*, τ_NSE_)/*S*(*Q*, 0) is visible. At *Q* = 0.18Å^-1^, a splitting occurs indicating a slight slowing down of the relaxation with increasing concentration of CalB.

## 6. Discussion

**Figure 5:**
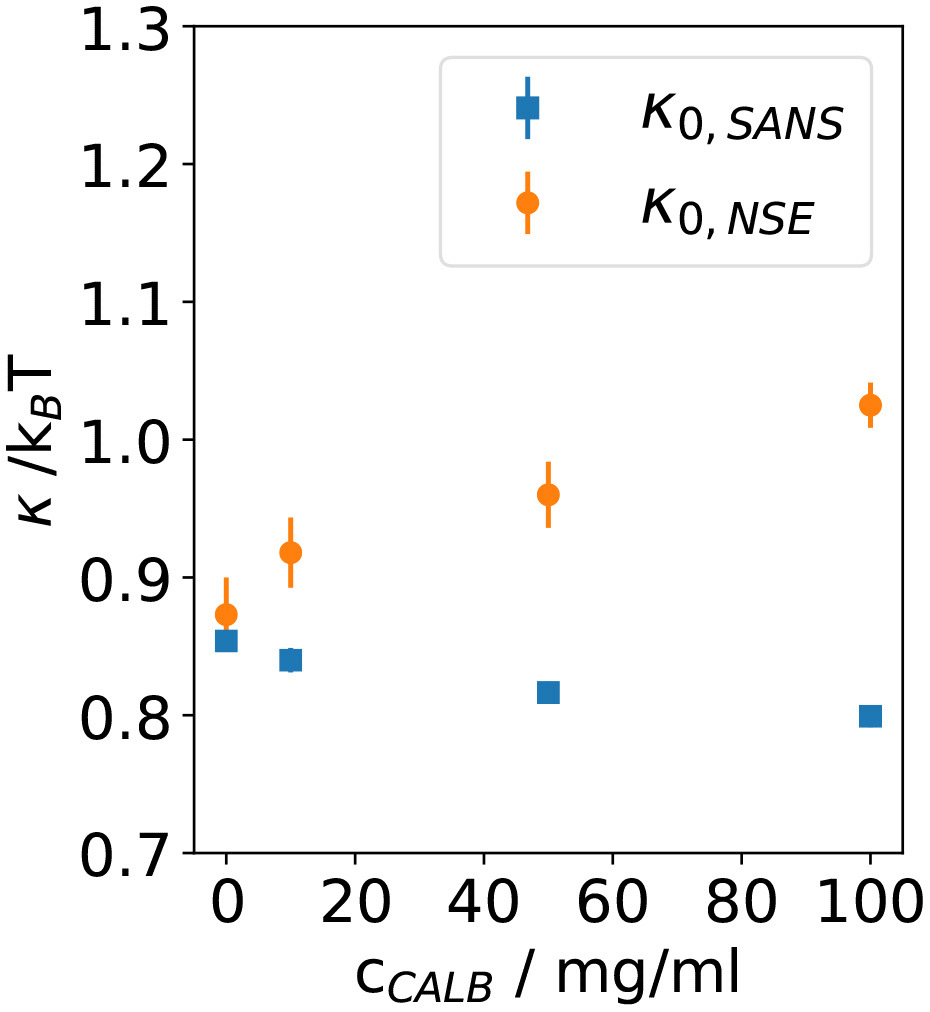
Bending rigidity constant κ_0_ obtained from fitting Equation (5) to the normalized intermediate scattering functions shown in Figure 1 for four samples with different lipase concentrations. Results from neutron spin echo (NSE) measurements are compared with the resulting values from analyzing the small angle neutron scattering (SANS) data.

In Figure 5, the bending rigidities κ_0,*SANS*_ and κ_0,*NSE*_ obtained from the fits to the SANS and NSE data are plotted as a function of the CalB concentration in the deuterated aqueous phase.

These results agree with previous theoretical and experimental work leading to the expectation of κ_0_ ≈ 1*k*_*B*_*T* for bicontinuous microemulsions. Without the addition of CalB, for the host microemulsion the SANS and NSE measurement yield κ_0,*SANS*_ ≈ κ_0,*NSE*_, which illustrates that the determination of the bending elasticity constants is independent from the method. After the addition of CalB, the values of κ_0,*SANS*_ slightly decrease from 0.85 to 0.8 *k*_*B*_*T* when more CalB was added. However, κ_0,*NSE*_ significantly increases from 0.9 to 1.1 *k*_*B*_*T* for the same concentrations of CalB.

SANS measurements time-average different configurations of a fluctuating and highly flexible sample structure over a time period of a few minutes. Usually, this counting time is required to achieve a sufficient signal to noise ratio of the recorded 2D scattering pattern.

In contrast to this, in soft matter samples, NSE inherently measures thermal fluctuations in the nanosecond time range at lengths up to a few ten nanometers.

In case of bicontinuous microemulsions, a SANS measurement averages innumerable topological arrangements of the highly flexible amphiphilic interfacial layer while NSE detects undulations and height fluctuations of this interface.

The lipase CalB consists of hydrophilic and hydrophobic parts. Hence, it is very likely that CalB can reside in the interfacial layer and act as a cosurfactant or amphiphilic block-copolymer. In this case, it is entirely possible that CalB changes the bending elasticity of the interface. Following the preceding argumentation, with SANS the time and space averaged bicontinuous structure was probed where CalB is either solubilized in the aqueous phase or resides as a cosurfactant or block-copolymer inside the amphiphilic interface. Moreover, if there were exchange processes, i.e., adsorption and desorption, between the aqueous phase and the interfacial layer, the SANS measurement includes an averaging of the two states.

**Figure 6:**
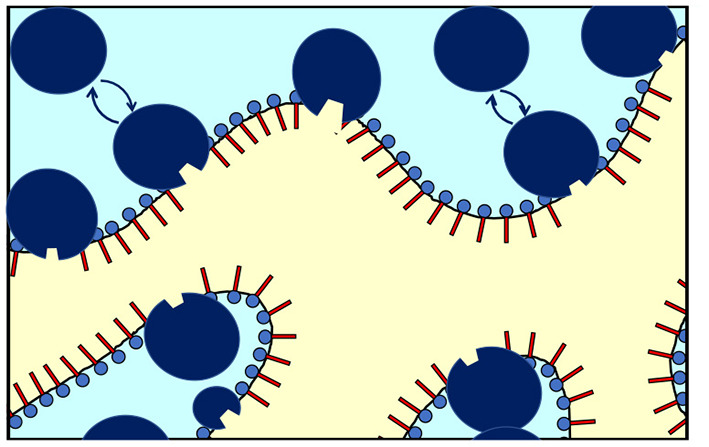
Schematic of the enrichment and exchange of *Candida antarctica* B (CalB) in the amphiphilic interface of a bicontinuous microemulsion with the surfactant layers and the aqueous phase (light blue) and the oil phase (yellow). The lipase CalB is assumed to reside temporarily at the interface. A fraction of the macromolecules is able to detach and return to the aqueous phase (in light blue). At the same time, the opposite process might take place to some extend (sizes of the surfactant and enzymes are not fully to scale).

The NSE measurement mainly detects fluctuations of the interface in presence of CalB. An effective increase of κ_0,*NSE*_ would result if CalB adsorbed to the interfacial layer causes a stiffening of the interface. However, in light of the phase studies, the local interfacial stiffening may be counterbalanced by an increasing amount of CalB in the water phase of the bicontinuous microemulsion that may induce a counter osmotic pressure as already discussed previously (Kabalnov et al., 1994; Subinya et al., 2014). Figure 6 sketches the presumed adsorption and desorption process of CalB at the interface.

This argumentation is in line with the previous publication showing that with increasing CalB concentration the concentration of both, the enzyme in the water phase and at the interface, increases (Subinya et al., 2014). Similar effects have also been observed in gradient polymers incorporated into microemulsion membranes, where the anchor point of the polymer may slide through the membrane and hence also lead to a variety of conformations on long length scales while being in one distinct conformation on the nanosecond time scale (Klemmer et al., 2017a,b). The enzyme modifies the bending rigidity and hence the free energy of the membrane, similarly as has been observed with the incorporation of diblock copolymers (Mihailescu et al., 2001) or the above-mentioned gradient polymers.

Contrary, in SANS and NSE measurements on the enzyme DFPase incorporated into the aqueous phase of a sugar surfactant-based microemulsion, there was no different behavior of the two bare bending elasticities. This spherically shaped enzyme shows no amphiphilicity and remains in the aqueous phase (Wellert et al., 2011). In conclusion, the results presented in the present paper in light of the previous phase studies allow for the following interpretation. Adding the lipase CalB to the water phase of a microemulsion of type H_2_O/NaCl–n-octane–C_10_E_5_ appears to induce two effects. First, the efficiency of the microemulsion runs through a maximum with the efficiency at high CalB concentrations leading to a decrease in efficiency even below the blank microemulsion, i.e., without CalB addition. Second, despite the efficiency running through said maximum value, the values of κ_0,*NSE*_ significantly increase from 0.9 *k*_*B*_*T* to 1.1 *k*_*B*_*T* for the same concentrations of CalB. While this appears to be counterintuitive at first, it is noted that both phenomena do not necessarily have to correlate as the underlying effects are different. While the observed trend in the efficiency may be attributed to an increase in osmotic pressure as a result of an increasing presence of CalB in the water phase, the significant increase in κ_0,*NSE*_ may be attributed to an increasing presence of CalB in the interfacial layer. Due to presumably fast adsorption and desorption processes, this effect can only be observed by NSE that provides access to the nanosecond time range at lengths up to a few ten nanometers. Consequently, if viewed locally, an increasing concentration of CalB in the water phase may also lead to an increase in bending stiffness, however, on a local scale in the vicinity of the adsorbed enzyme. The effect of CalB on the pure (NSE) bending rigidity seems to be especially pronounced already at low concentrations of CalB, if saturation effects occur and the change in κ is slowed down with increasing CalB concentration remains speculative with the present set of experiments. Keeping these two effects in mind, the application of CalB-catalyzed reactions involving non-natural substrates may thus be determined by a counterbalance between efficiency and interfacial access of the lipase. While solubilization of a non-natural substrate is a matter of efficiency, yield on the other hand may be a matter of interfacial access. Future investigation would need to show as to whether efficiency or interfacial access should be preferred in lipase-catalyzed reactions involving non-natural substrates.

## 7. Conclusion

In this study, we have studied how the introduction of the lipase CalB alters the bending elastic properties of the amphiphilic interface in a bicontinuous microemulsion of the ternary system D_2_O/NaCl–n-(d)-octane–pentaethylene glycol monodecyl ether (C_10_E_5_). We have determined the renormalized bending elastic constant κ_0_ using two scattering techniques. We determined κ_0, SANS_ from SANS and κ_0, NSE_ from NSE.

The results of both types of measurements show differences when the concentration of CalB is increased up to 100 mg/ml. While no significant influence of adding CalB to the aqueous phase of the microemulsion is observable for κ_0, SANS_, κ_NSE_ increases when the concentration of CalB rises. This indicates a stiffening of the amphiphilic interface. We attribute this effect to an increasing adsorption of CalB at the amphiphilic interface of the bicontinuous microemulsion. The difference between the structural parameters extracted from SANS on longer times and from NSE, detecting the local short time fluctuations, suggests that the lipase CalB temporarily adsorbs to the interface. We assume the respective residence time to be longer than the typical observation time of NSE of a few 10 ns. The complex situation of this dynamical equilibrium with the lipase being partly adsorbed at the membrane for some time, partly dissolved in the aqueous phase, with the details of the interaction of the lipase with the membrane not exactly known, requires more investigations, for example with molecular dynamics simulations, to shed more light into the lipase-membrane interaction.

The results from NSE experiments are in line with previous results (Klemmer et al., 2017a,b), suggesting that the approach of measuring the elastic constants at different time scales leads to information on the interaction of the additives with the microemulsion membrane. In the case of lipase CalB, the residence time at the membrane at the oil–water interface might be an important parameter for catalytic activities, in particular regarding non-natural substrates.

## Data Availability Statement

The raw data supporting the conclusions of this article will be made available by the authors, without undue reservation.

## Author Contributions

SE and SW: conception and idea. SE, SR, BNes, and BNeb: sample preparation and phase diagram measurements. BH, SE, SW, HF, and OH conducted the neutron scattering experiments. Data evaluation and writing of the paper by SE, SW, and OH. All authors contributed to the article and approved the submitted version.

## Conflict of Interest

The authors declare that the research was conducted in the absence of any commercial or financial relationships that could be construed as a potential conflict of interest.
