# Supplementary figures and images for: Surfactant Monolayer Bending Elasticity in Lipase Containing Bicontinuous Microemulsions
Source: Front Chem. 2021 Jan 5;8:613388. doi: 10.3389/fchem.2020.613388 (PMC7814304; doi:10.3389/fchem.2020.613388)

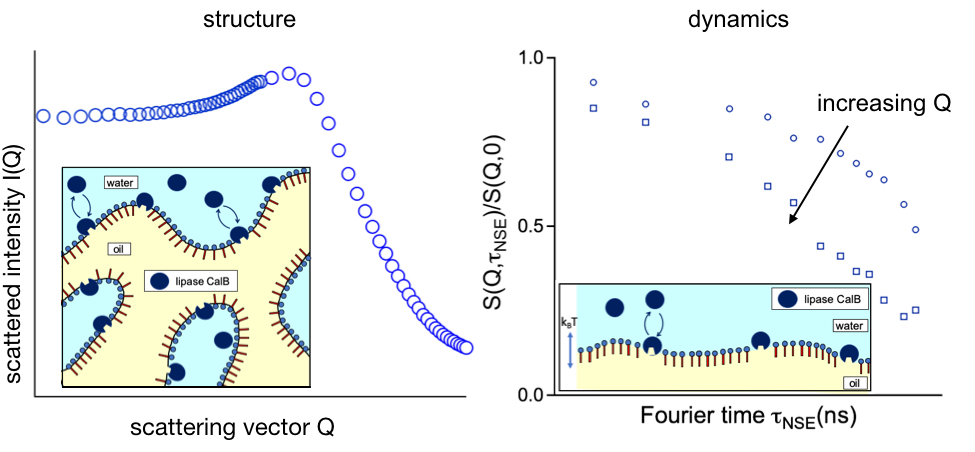

Supplement: Supplementary file 1 [file Image_1.JPEG]
